# Supplementary figures and images for: Coadaptation of the chemosensory system with voluntary exercise behavior in mice
Source: PLoS One. 2020 Nov 25;15(11):e0241758. doi: 10.1371/journal.pone.0241758 (PMC7688120; doi:10.1371/journal.pone.0241758)

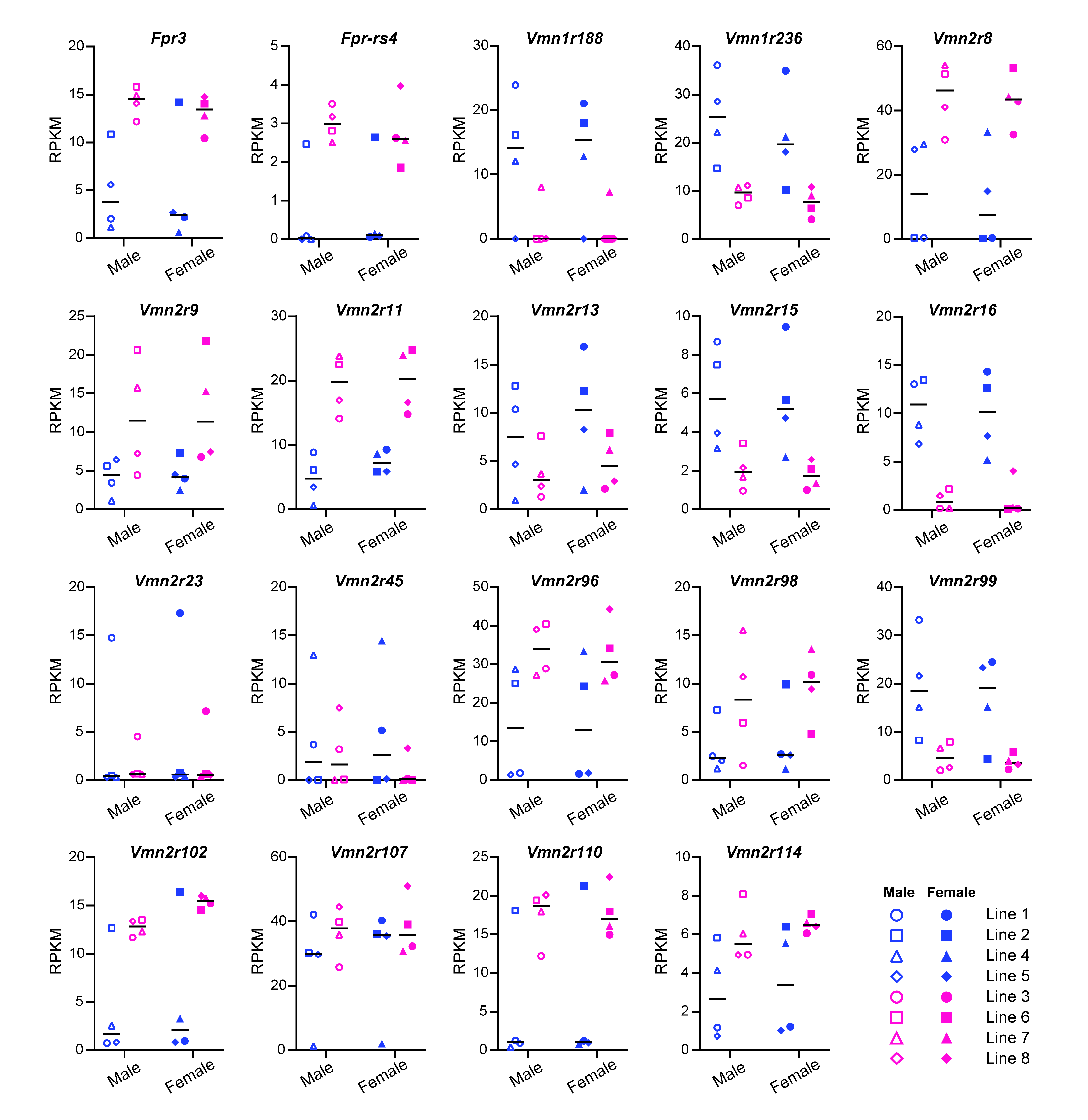

Supplement: S1 Fig — Scatter plots showing the RPKM of DE vomeronasal receptor genes in males and females from each line of HR or Control mice. Black bars represent medians. (TIF) [file pone.0241758.s001.tif]

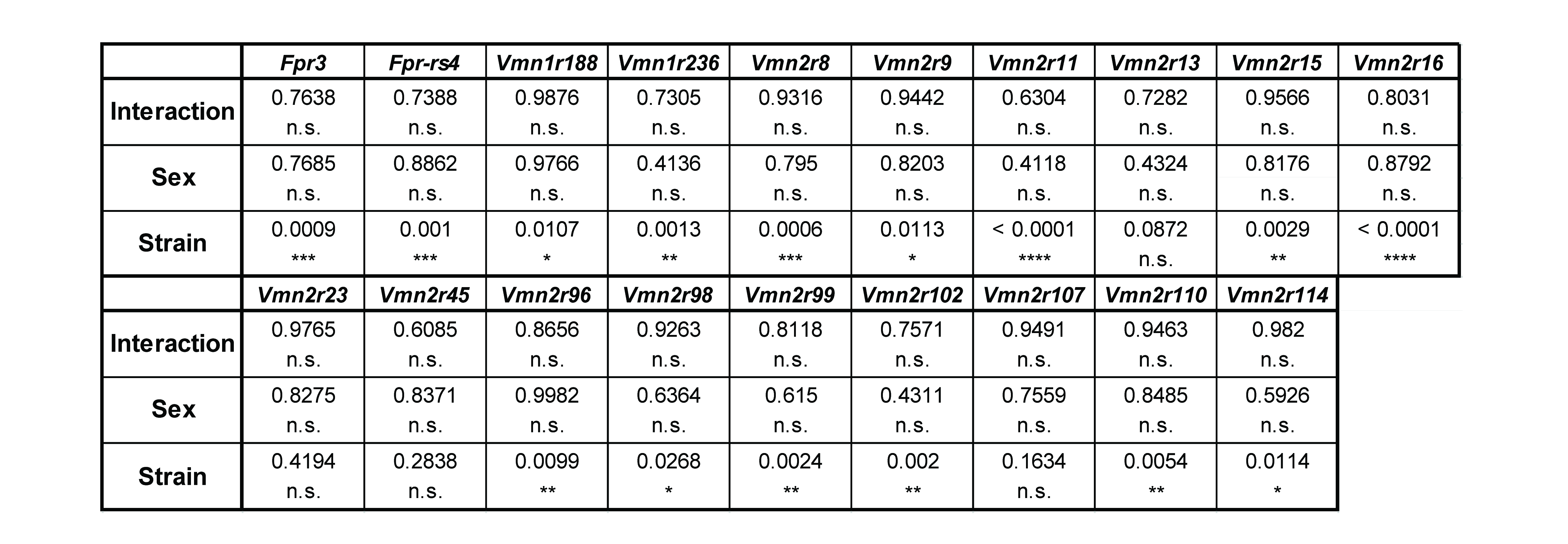

Supplement: S2 Fig — A table showing p-values for interactions, sex differences, and linetype differences in the RPKM of DE vomeronasal receptor genes in two-way ANOVA analyses. n.s., *, **, ***, and **** represent not significant, p < 0.05, p < 0.01, p < 0.001, and p < 0.0001, respectively. (TIF) [file pone.0241758.s002.tif]

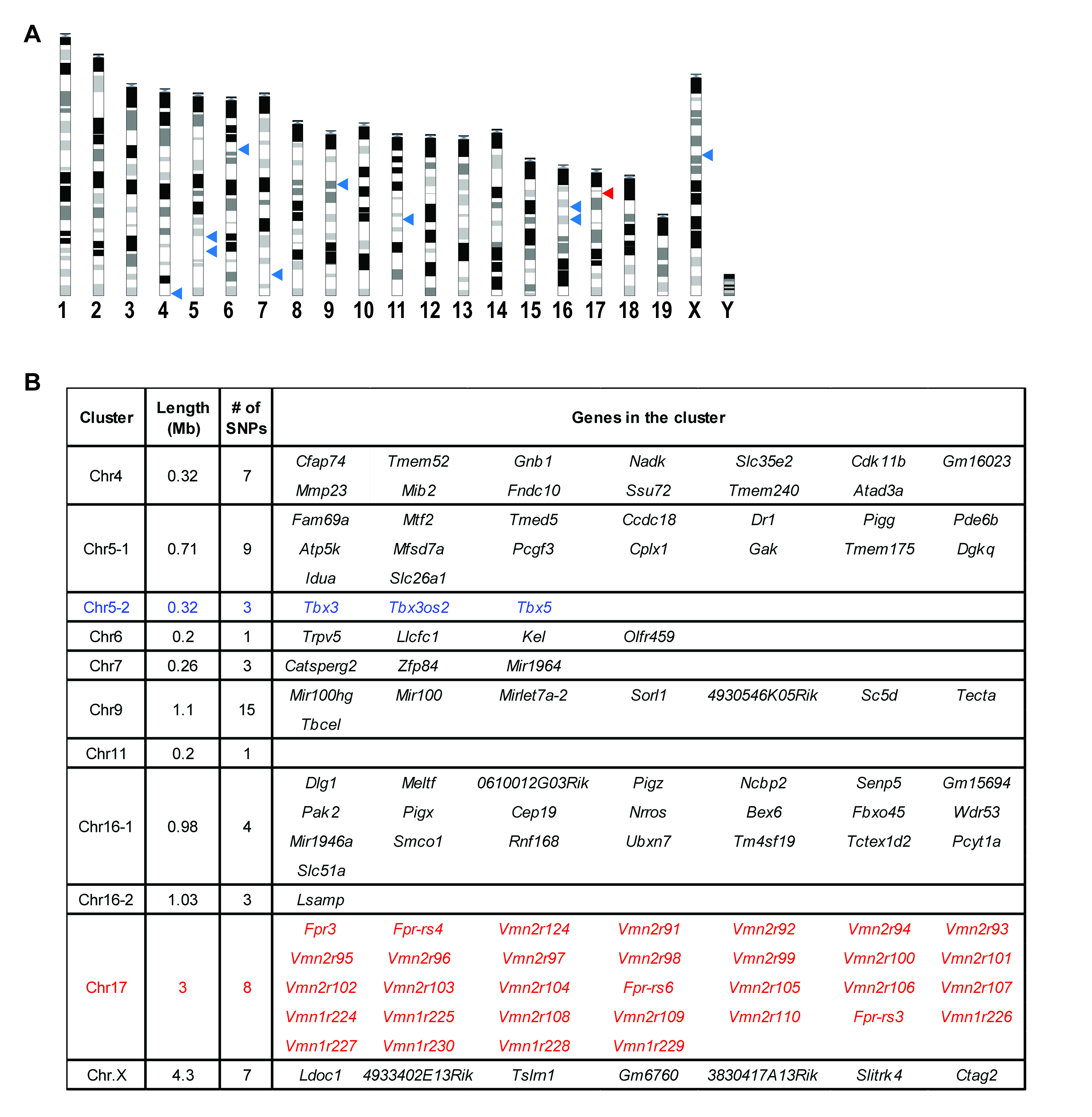

Supplement: S3 Fig — (A) A schematic diagram showing the relative positions of loci containing 1 or more all-or-none SNPs. Blue triangles indicate non-chemosensory clusters, and a red triangle indicates clusters containing only chemosensory (vomeronasal) receptors. (B) A table showing chromosomal location and length of each all-or-non SNP cluster, and the number of SNPs and genes within the clusters. The row highlighted in red is the cluster containing only the vomeronasal receptor genes. (TIF) [file pone.0241758.s003.tif]
